# Alerting, orienting or executive attention networks: differential patters of pupil dilations

**DOI:** 10.3389/fnbeh.2013.00145

**Published:** 2013-10-14

**Authors:** Ronny Geva, Michal Zivan, Aviv Warsha, Dov Olchik

**Affiliations:** ^1^Department of Psychology, Bar Ilan UniversityRamat Gan, Israel; ^2^Brain Sciences program, The Gonda Multidisciplinary Brain Research Center, Bar Ilan UniversityRamat Gan, Israel

**Keywords:** arousal, attention, autonomic response, pupil dilation, Norepinephrine, locus coeruleus

## Abstract

Attention capacities, alerting responses, orienting to sensory stimulation, and executive monitoring of performance are considered independent yet interrelated systems. These operations play integral roles in regulating the behavior of diverse species along the evolutionary ladder. Each of the primary attention constructs—alerting, orienting, and executive monitoring—involves salient autonomic correlates as evidenced by changes in reactive pupil dilation (PD), heart rate, and skin conductance. Recent technological advances that use remote high-resolution recording may allow the discernment of temporo-spatial attributes of autonomic responses that characterize the alerting, orienting, and executive monitoring networks during free viewing, irrespective of voluntary performance. This may deepen the understanding of the roles of autonomic regulation in these mental operations and may deepen our understanding of behavioral changes in verbal as well as in non-verbal species. The aim of this study was to explore differences between psychosensory PD responses in alerting, orienting, and executive conflict monitoring tasks to generate estimates of concurrent locus coeruleus (LC) noradrenergic input trajectories in healthy human adults using the attention networks test (ANT). The analysis revealed a construct-specific pattern of pupil responses: alerting is characterized by an early component (Pa), its acceleration enables covert orienting, and executive control is evidenced by a prominent late component (Pe). PD characteristics seem to be task-sensitive, allowing exploration of mental operations irrespective of conscious voluntary responses. These data may facilitate development of studies designed to assess mental operations in diverse species using autonomic responses.

## Introduction

Alerting responses; orienting to sensory stimulation; and monitoring of thoughts, actions, and emotions play integral roles in multiple psychological and psychopathological processes, including target detection in complex environments and in monitoring processes. These operations are important in regulating the behavior of multiple species along the evolutionary ladder (Romberg et al., 2013). Alerting to non-specific cues has long been documented in multiple species. Orienting of attention has also been observed in multiple models, including rat (Hopkins et al., 2009) and macaque (Bowman et al., 1993), and executive monitoring was reported in rhesus macaques by using the numerical Stroop effect (Washburn, 1994; Yoshida et al., 2012)

Exploring such mechanisms in self and in others is considered important, as they underlie observational learning and allow adaptive behavior (Yoshida et al., 2012). Yet to date, differentiating among alerting, orienting, and executive control monitoring is a challenge in behavioral neuroscience. There is a need to develop non-invasive objective techniques to distinguish among these cognitive operations irrespective of voluntary performance. One potential avenue toward this endeavor may be a deeper exploration of autonomic-cognitive relations (Tursky et al., 1969). Each of the primary attention constructs—alerting, orienting, and executive control monitoring—involves salient autonomic correlates as evidenced by changes in reactive pupil dilation (PD, Gilzenrat et al., 2010; Gabay et al., 2011; Nassar et al., 2012), heart rate (Richards and Casey, 1991), and skin conductance (Frith and Allen, 1983). Earlier work with these indices pointed to the efficacy of using PD as the best single autonomic indicator of attentive mental effort (Kahneman, 1973), yet its temporo-spatial characteristics in each attentive state are not known. Recent technological advances in recording PD at high resolution introduces the potential to examine autonomic temporo-spatial differences in order to tell alerting, orienting, and executive control apart by reading PD patterns.

### Psychosensory PD response

Somatic and visceral afferents for sensory, motor, and internal motor operations, as well as all central connections related to arousal responses, can trigger the psychosensory PD reflex (Bradley et al., 2008), whereas light accommodation elicits restriction responses. Recent high-impact findings describe pupillary response involvement in specific attention networks, including arousal (Gilzenrat et al., 2010), orienting (Gabay et al., 2011), and effortful control operations (Nassar et al., 2012). Each of these lines of evidence provides much needed support for interpretations of otherwise similar looking data (Bijleveld et al., 2010). However, given the experimental and data analyses methods used, it is difficult to attribute autonomic responses to individual attention networks.

Research points to the locus coeruleus (LC), the sole source of noradrenergic neurons in the brain, as playing a key role in cognitive processes (for recent reviews; Sara, 2009; Laeng et al., 2012), including preconscious preparation (Laeng et al., 2012), attention (Corbetta et al., 2008), sensory processing, memory formation, memory retrieval (Sterpenich et al., 2006), decision making, and performance facilitation (Laeng et al., 2011), and network resetting (Dayan and Yu, 2006; Sara, 2009). Its versatile involvement, particularly in cognitive contexts that require regulation of arousal and management of high loads, implies that its activity is involved in mediating each of the three attention networks.

The LC is also known to be highly involved in PD regulation. Single-cell recordings in monkeys provided evidence of a strong correlation between PD and LC–norepinephrine (NE) neuron activity (Rajkowski et al., 1994). It is known that PD reflects NE release from the LC (Koss, 1986; Bremner and Smith, 2001), and this event moderates arousal by activating inhibitory α_2_-adrenoceptors in the Edinger–Westphal nucleus, which is a parasympathetic preganglionic region (Samuels and Szabadi, 2008).

Recent technological advances have enabled the use of direct, on-line, gaze, and pupillary reactivity tracking in human attention studies that allow researchers to distinguish temporal and spatial characteristics of autonomic/voluntary interplay that uniquely characterize individual attention networks (Laeng et al., 2011; Laeng et al., 2012).

However, it remains unclear how alerting, orienting, and/or executive responses can be differentiated with PD. Aston-Jones and Cohen's gain theory (Aston-Jones and Cohen, 2005; Gabay et al., 2011) alluded to two different modes of LC–NE activity, tonic and phasic, which prime two fundamental and dissociable cognitive mechanisms. According to this theory, LC neurons exhibit a tonic activity mode, associated with transition to a new task, disengagement from the current task, and a search for alternative behaviors (exploration), while phasic LC activation is driven by the outcome of task-related decision processes and is proposed to facilitate ensuing behaviors and to help optimize task performance (exploitation). This framework may imply that a non-specific alerting cue or the absence of a specific cue would elicit an exploration LC mode driven by an expectation to respond quickly to a target, while executive monitoring of incongruent stimuli, as in Stroop-like tasks would elicit an exploitation phasic LC mode (Laeng et al., 2012) to enable effortful monitoring of more demanding tasks (Gabay et al., 2011). *The current study attempts to extend this framework to encompass all three attention networks by using PD to examine the construct-specific temporal and spatial LC*–*NE system correlate to characterize all three attention networks*. On the basis of PD research pertaining to alerting, orienting, and executive control, we formed three discrete yet complementary construct-specific hypotheses.

### Alerting PD hypothesis

Alerting (readiness to receive information) and subsequent activation (readiness to respond) are core self-regulatory mechanisms present even in neonates that are considered homeostatic processes that regulate an energetic pool (Tellinghuisen et al., 1999) as a function of cognitive load (Geva et al., 1999) and emotional arousal (Hoehl and Striano, 2009). They allow for sensitivity to (Rose et al., 2005; Harel et al., 2010) and readiness for the reception of novel non-specific stimuli (Tellinghuisen et al., 1999; Colombo, 2001) by tonic changes in activity.

Arousal is related to hindbrain noradrenergic mechanisms (Rajkowski et al., 1994) through relays from the LC that, in addition to other reticular formation structures, serve as a primary neural substrate for arousal and further activation in the thalamus and parietal and frontal cortices to enable an alert response (Tracy et al., 2000). The adaptive gain theory highlights the pivotal role of the LC–NE system in regulation and attention (Robbins, 1984) during engagement and vigilance (Aston-Jones, 2005) and for optimizing attentiveness (Howells et al., 2012). Its activation by a non-specific warning cue leads to the replacement of resting state with a new state involving preparation for detecting and responding to an expected signal (Petersen and Posner, 2012). We therefore suggest that presentation of non-specific alerting cues in the environment would activate an LC activity compatible with an exploration mode relative to conditions without such warning signals. The change in response to a relatively non-specific cue, termed Pa, would be (a) evidenced by a gentle onset rise after cue presentation (~150–200 ms; Aston-Jones, 2005); (b) apparent before the eyes move to the target, i.e., preconsciously (Laeng et al., 2012); and (c) sustained throughout the task after conscious decision execution (Aston-Jones et al., 1994).

### Orienting PD hypothesis

The orienting network directs attention to a target stimulus. This network can be triggered by specific spatial cues, as well as cues in other modalities. The orienting response is considered a product of a distributed neural network, which includes the frontal eye fields (Wardak et al., 2006), the superior parietal lobe and temporal–parietal junction (Fuentes and Campoy, 2008), superior colliculus, and the pulvinar nucleus of the thalamus (Shipp, 2004).

Orienting can be achieved with (overtly) or without (covertly) eye movement toward a target location. LC–NE-related PD changes in response to salient environmental stimuli (Rajkowski et al., 1994), such as activating reactivity to a specific cue in space that precedes the target (Petersen and Posner, 2012). The autonomic orienting response activation to a valid specific cue, as compared with a non-specific one, is expected to enable *acceleration of the onset and rate of enhancement of the alerting response, Pa* (Stelmack and Siddle, 1982; Callejas et al., 2004). Thus, LC–NE input would enable a more efficient orienting response (relative to the non-specific alerting response) by providing additional input to speed up mental resource recruitment, resulting in a shorter and steeper onset time for Pa compared with the onset of Pa in response to a non-specific alerting cue. At the same time, the valid specific spatial cue is not necessarily expected to be more alarming than a non-specific cue and is therefore not expected to elicit a greater Pa amplitude increase (Steiner and Barry, 2011).

### Executive control monitoring PD hypothesis

The executive attention network for error monitoring is associated with mental resource recruitment to manage cognitive load (Van Steenbergen and Band, 2013), such as is required to resolve conflicts and act contrary to one's habits/expectations (Fan et al., 2005). Initial work suggested that PD may be the most useful autonomic indicator of mental effort and that PD is the best single index of such an effort (Colman and Paivio, 1969; Tursky et al., 1969). This line of evidence was later extended by showing that moment-by-moment changes in mental effort correlate with PD in a dose–response manner (Karatekin, 2004) to enable the suppression background information and/or familiar expectations (Nassar et al., 2012). This response, termed Pe, is thought to result from cortical modulation of the reticular formation (Steinhauer et al., 2004) through activation of a top–down control network involving the medial–ventral prefrontal cortex, anterior cingulate cortex (ACC), and lateral prefrontal cortex executive network (Bush et al., 2000; MacDonald et al., 2000).

The difference in the distributed neural networks involved in the alerting and orienting networks as compared with the executive effortful one may indicate that spatiotemporal attributes of Pe should to be different than those of the Pa component. Pe is expected to reflect a different LC activity mode than Pa. Ashton-Jones and Cohen's gain theory suggested a dual mode of LC activity, where the LC–NE system is activated in the phasic mode during more demanding tasks. Such a phasic PD response has been shown during a Stroop distraction task, with a long delay of the order of 1400 ms post-stimuli presentation (Laeng et al., 2011). The delay is thought to be due to the activation time needed to modulate autonomic arousal through top-down pathways that originate in cortical areas, e.g., prefrontal and cingulate cortex (Matthews et al., 2004), and spreading through the brainstem and LC to induce measurable changes in pupil size. Therefore, we hypothesized that executive tasks entailing effortful monitoring while suppressing distracting information should evoke *prominent temporally delayed* responses (Pe). This should be in addition to the early emerging Pa alerting component.

The *magnitude of Pe should represent a dose–response relationship* based on the invested effort. With this latter notion in mind, it is hypothesized that Pe should be augmented as a function of incongruency rather than mere incompatibility among stimuli, particularly in unfamiliar, less expected trials with incongruency compared with well-practiced ones.

In summary, on the basis of previous findings regarding pupillary responses pertaining mostly to specific attention networks, we propose that distinct pupillary responses should be expressed in all three attention networks in a temporo-spatial construct-specific manner that would allow differentiation between alerting, orienting, and executive control monitoring responses by measuring dynamic changes in PDs. Specifically, we hypothesize that arousal PD responses would be compatible with early mild PD changes and would be maintained throughout the trial. An orienting response would be characterized by the same response as arousal, but with an accelerated onset. Finally, executive control monitoring response would be corroborated by a prominent phasic change in the PD response, which would be delayed and locked to the decision response, with amplitude proportional to momentary mental effort. We tested these hypotheses within the Attention Networks framework (Fan et al., 2002; Petersen and Posner, 2012).

## Materials and methods

### Participants

Twenty-seven healthy young adults (age = 26.7 ± 4 years, 40% female) with normal intelligence (WAIS III short-form IQ = 115 ± 4) participated in the study. Volunteers were excluded if they had received a diagnosis of attention deficit hyperactivity disorder (ADHD). Additionally, all participants reported up to one positive item on the inattention and hyperactivity portions of the ADHD-DSM questionnaire, no anxiety on the State-Trait-Anxiety Inventory (mean: 29 ± 5), no depression on the Beck questionnaire (group mean: 3.3 ± 0.6), and normal or corrected-to-normal vision. Participants confirmed that they had not taken drugs, alcohol, or medication on the day preceding testing.

### Stimuli

**Figure 1:**
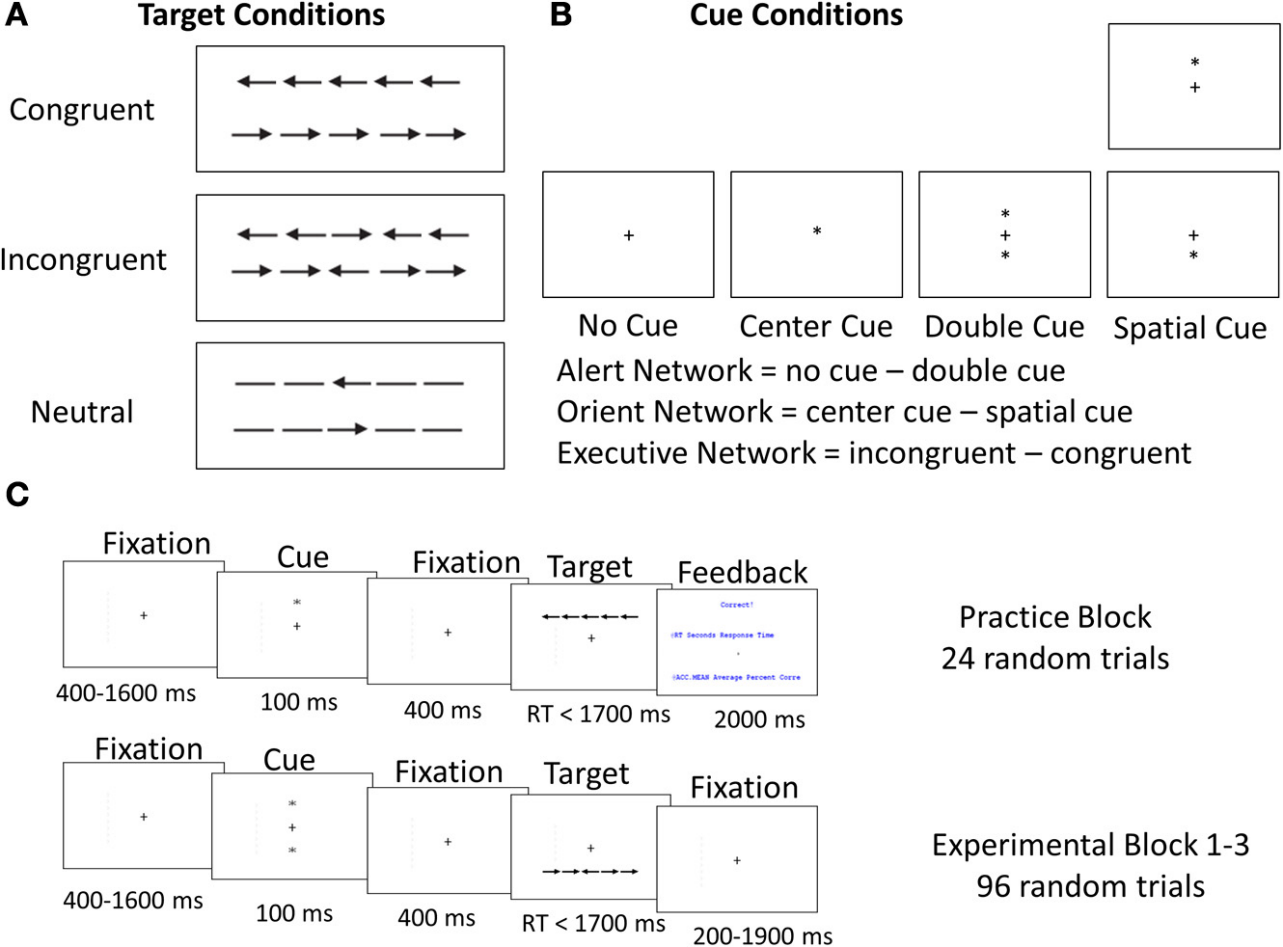
**Attention Network Task (ANT). (A)** Target conditions, **(B)** Cue conditions, **(C)** Experimental protocol.

The Attention Network Task (ANT), a theoretically derived test, was developed to test alerting, orienting, and executive control networks (Petersen and Posner, 2012) using a within-subject repeated-measures design with seven experimental conditions. The ANT is widely used to measure reactivity to visual stimuli and allows for comparisons among the three attention networks (Fan et al., 2002). It is neuroanatomically validated (Konrad et al., 2005) and uses orthogonal manipulations to examine each network (Fuentes and Campoy, 2008). The ANT is comprised of seven discrete conditions consisting of combinations of cued reaction time tasks and flanker tasks. The tasks themselves consist of four cue conditions (no cue, center, double, and orienting) and three target flanker conditions (congruent, incongruent, and neutral; Figure 1).

The stimuli consisted of a row of five arrows pointing left or right, which appeared in one of two positions on the monitor (upper, lower). The target stimulus was the middle arrow. Different cues were presented to test the alerting network (no cue before the target vs. double cue) and orienting network (central cue vs. spatial cue). The conflict network was tested by presenting different arrow flankers on both sides of the target stimulus (two on each side). The flankers were either incongruent with the target-direction (pointing in the opposite direction), congruent (identical to the target), or neutral (no specified direction). Using an external computer mouse, participants were directed to press the left or right buttons according to the direction of the target. During the task, a fixation cross was replaced by a cue, which informed the subject when and where the arrow would appear (Figure 1).

This paradigm is comparable with the canonical ANT operational model offered by Fan et al. (2002) for testing the efficacy and independence of the attention networks using the ANT. It typically analyzes differences between conditions that exemplify the underlying construct of each network. The alerting network contribution (i.e., presence or absence of cues without spatial information) was deduced by computing the difference between trials that were preceded by a double cue and those that had no cue. The orienting network contribution (i.e., presence or absence of cues with spatial information), was calculated by computing the response difference between trials that were preceded by spatial cues and those with central cues. The executive network contribution was measured by the differences between congruent flankers on both sides of the target and incongruent flankers. This network also has a third control condition with neutral flankers, which allowed us to assess differences in performance as a function of incompatible stimuli compared with incongruent ones.

### Procedure

Participants underwent the ANT using the Tobii 1750 binocular eye tracker (Tobii Technology AB, Danderyd, Sweden), which records eye movements and PDs (Tobii Technologies, 2006). Testing was conducted in a quiet room enclosed by a gray curtain. Luminance levels in each condition were all 340 lux as measured using a Lux light meter (model LX-1010BS), 50 cm away from the screen at the height of the cues, perpendicular to the screen. Taking into account this model's reported sensitivity at this luminance level, the expected error measurements are small, on the order of ±13.6 lux, which limits the concern of inter-stimuli luminance difference. Gaze behavior was monitored throughout the trial on a separate monitor behind the curtain to ensure continuous data recordings.

The experiment consisted of four blocks. The first practice block took about 2 min, the other three experimental blocks lasted about 5 min each, with the entire experiment running about 20 min. Participants were required to decide the middle arrow's direction (left or right) and to respond as quickly as possible by clicking the corresponding mouse button. An ANT session consisted of 24 practice trials (not analyzed) and three experimental blocks with 144 Alerting and Orienting network trials and 192 Executive-Control network trials. Participants were allowed a short break between blocks. Each trial consisted of five events: a fixation period with a center cross (ranging from 400 to 1600 ms), followed by a 100-ms flashing star warning cue (no cue, double cue, spatial cue, or central cue). After another 400-ms fixation period, the stimulus appeared and remained on the monitor until the participant responded, up to 3500 ms (Figure 1, lower panel). Behavior-dependent measures included response times (RT) and errors in each trial.

### Gaze tracking

We employed a 2.66-GHz Core 2 Duo PC integrated with a Tobii 1750 binocular eye tracking system and near infrared diodes to generate reflections on participants' corneas. These reflection patterns and other visual information were collected using a camera. The system tracks both eyes to a rated accuracy of 0.5°, sampled at 50 Hz. The system was successfully calibrated for each participant using a 5-point calibration.

#### Data acquisition and analysis

E-prime 2 software (Psychology Software Tools, Inc. Sharpsburg, PA) was used to present the experiment, and all eye gaze positions and PD data at 50-Hz sampling rates were recorded by the eye tracker as participants viewed specified cues and target areas of interest (AOIs). Gaze-dependent measures included latency to fixate on all cues and target AOIs, continuous PD recordings, use of the E-prime clock to synchronize button presses, and gaze data sources.

### PD reactivity

To analyze PD patterns in each trial condition, a baseline for each eye was initially calculated by averaging the PD recordings of the 200 ms preceding each trial (fixation period), during which time a fixation point appeared on the screen and no response was required, causing PD to be at its lowest values for this experiment. Luminance at baseline was comparable to that during the cue presentation period. The baseline value was subtracted from the PD recordings for each eye during a fixed 2100-ms period of each trial (500 ms before target onset—cue onset). Trials were synchronized using the target onset as time zero and then classified according to the different conditions. PD peaks were manually identified by viewing the graphs for each participant in each condition.

#### Missing data

**Figure A1:**
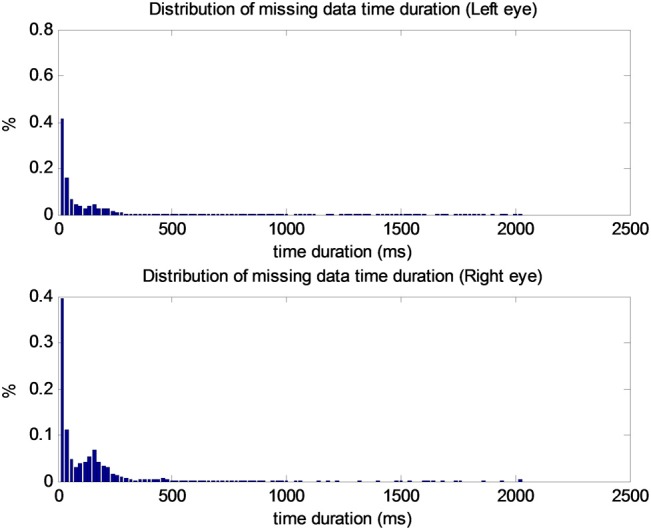
**Distributions of missing data time durations**. Left eye (**top**), right eye (**bottom**).

Missing data periods are expected due to blinking and periods in which participants look away from the monitor. Trials with more than 50% missing data in both eyes were excluded from the analysis (3.11% of trials), and 11.74% of data were missing overall. The distribution of lengths of missing data periods for each eye is depicted in Figure A1, in which the *X*-axis represents missed data duration and the *Y*-axis displays the percentage of missing data for the total time. The figure shows that most missing data periods (almost 70%) lasted less than 80 ms. The relative scarcity of missing data in this dataset and the short durations of missing data periods limit concern with regard to data interpolation bias.

#### Data interpolation and filtering

Missing data were filtered using a low-pass digital filter and interpolated based on Jackson and Sirois (2009) method, which was applied forward and backward to prevent phase drift using the following equation:

$$y(n)=a_{0}x(n)+a_{1}x(n-1)+a_{2}x(n-2)+b_{1}y(n-1)+b_{2}y(n-2),$$

where *a*_0_ = *a*_2_ = 0.0336, *a*_1_ = 2^*^a_0_; *b*_1_ = 1.419, and *b*_2_ = −0.533.

#### Data interpolation phase

Missing data for only one eye were interpolated using baseline-adjusted data from the other eye. Linear interpolation was applied by averaging the three samples before and after the break. Finally, left and right PDs were averaged.

#### Network computations

Each network had a baseline cue condition (e.g., no-cue, central cue, and congruent cue conditions) and a network-specific cue condition (double cue, spatial cue, and incongruent cue conditions). The network-dependent differences for each network were calculated as follows (*N* = number of trials, *M* = number of participants):

$$\begin{array}{l} \text{Alerting network:}\\ \frac{\sum_{i=1}^{M}\sum_{j=1}^{N}{\left(\text{No Cue RT}-\text{Double Cue RT}\right)}_{i,j}}{M*N}\\ \text{Orienting network:}\\ \frac{\sum_{i=1}^{M}\sum_{j=1}^{N}{\left(\text{Central Cue RT}-\text{Spatial Cue RT}\right)}_{i,j}}{M*N}\\ \text{Executive Control Network:}\\ \frac{\sum_{i=1}^{M}\sum_{j=1}^{N}{\left(\text{Incongruent flankers RT}-\text{Congruent flankers RT}\right)}_{i,j}}{M*N} \end{array}$$

## Results

### Dynamic pupillary activation in the three networks

To characterize the trajectory evoked in each network as a function of condition (baseline vs. cue), a series of paired *t*-tests with a significance level of *p* < 0.0005 was performed for each network as a function of condition to trace each 20-ms period during the trial. All relevant trials were aligned according to target onset. Results showed two distinct pupillary responses in each network: an early peak (Pa) preceding the response, and a later higher peak (Pe) that occurs about 600 ms after the response. Pa and Pe amplitudes were calculated as the difference in PD in Pa and Pe, respectively, relative to PD at the time of the cue onset, which marks the beginning of the active phase of the trial.

**Figure 2:**
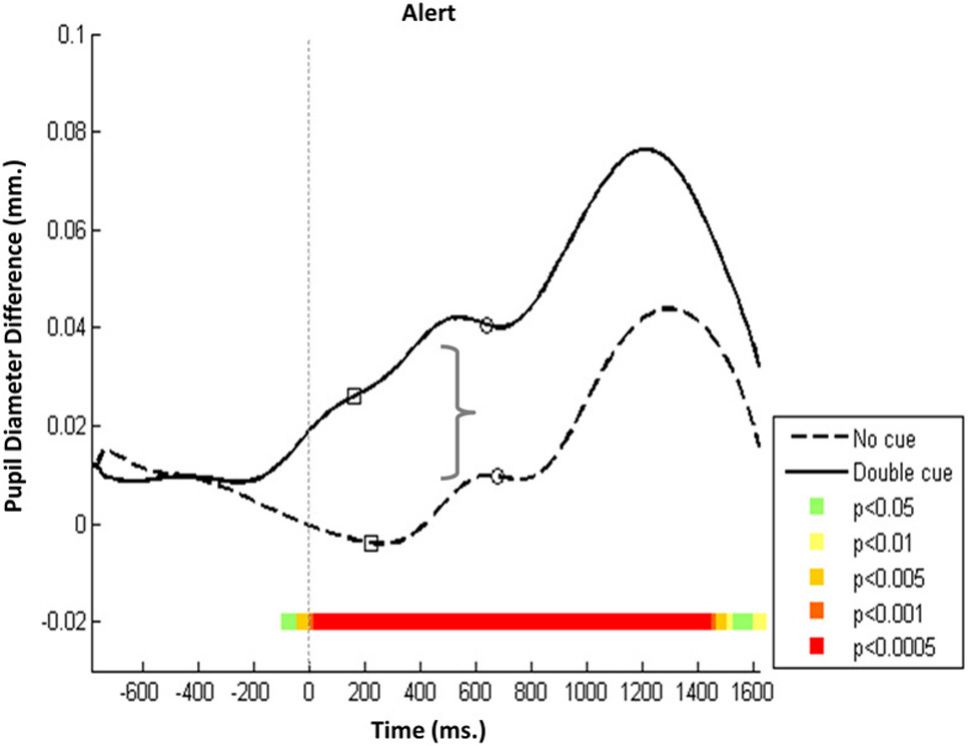
**Alerting network—Mean PD dilation**. Blue line, no cue trials; black line, double cue trials; white squares, mean entrance to target AOI; white circles, mean button press. Time zero represents target onset (dashed gray line) and −500 ms cue onset (dotted gray line).

Pa and Pe peak characteristics were network specific. In the alerting network, Pa initiation in the double-cued trials preceded initiation in the no-cue condition and was seen as early as 300 ms after the cue. This initiation indicated an augmented response throughout the double-cue condition at a significantly higher level than for the no-cue condition (Figure 2, *p* < 0.0005). The amplitude of the Pa component was larger in the double-cue condition than in other less saliently cued conditions (i.e., non-cue, spatial cue, and central cue conditions). This finding supports the hypothesis that Pa might be related to an arousal mechanism operated by the alerting network.

**Figure 3:**
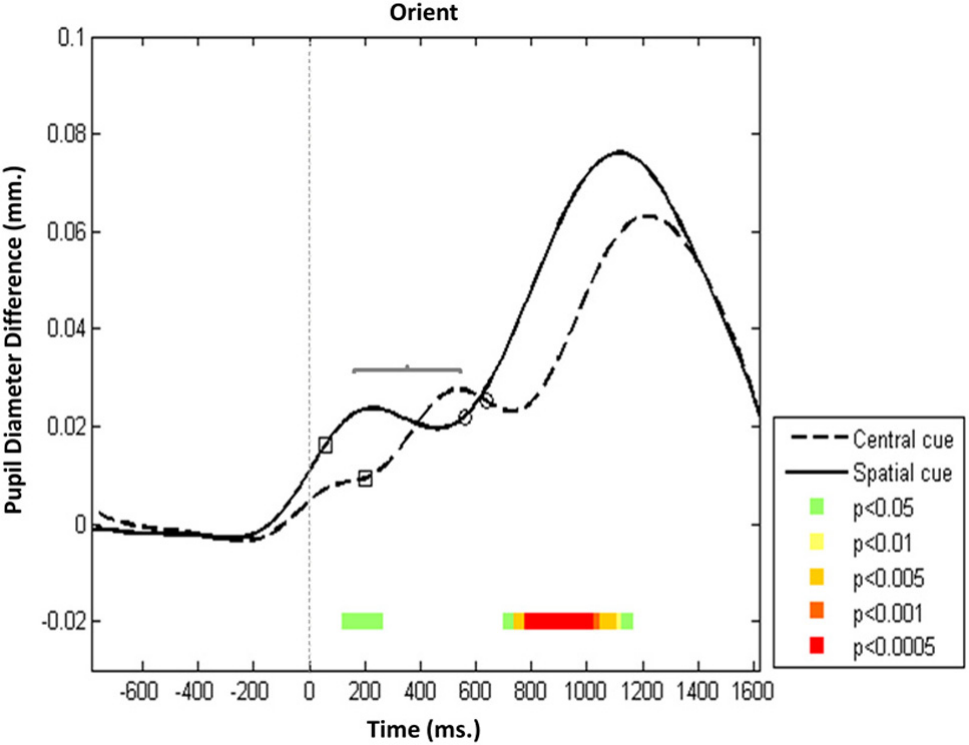
**Orienting network—Mean PD dilation**. Blue line, central cue trials; black line, spatial cue trials; white squares, mean entrance to target AOI; white circles, mean response time. Time zero represents target onset (dashed gray line) and −500 ms cue onset (dotted gray line).

The same overall trajectory was observed in the orienting network (Figure 3), but there were significant differences in the timing of onset and the acceleration rate of pupillary reaction between the two conditions (central vs. spatial cue). In the spatial condition, Pa was evoked and its latency to peak was, on average, 200 ms earlier than in the central cue condition (*p* < 0.05), but the amplitude of Pa was stable across these orienting conditions. This finding supports the notion that selectively orienting gaze to a specific cue in space is preceded by acceleration in P1 activation relative to conditions that lack a specific orienting cue (i.e., non-cue, double cue, and central cue conditions).

**Figure 4:**
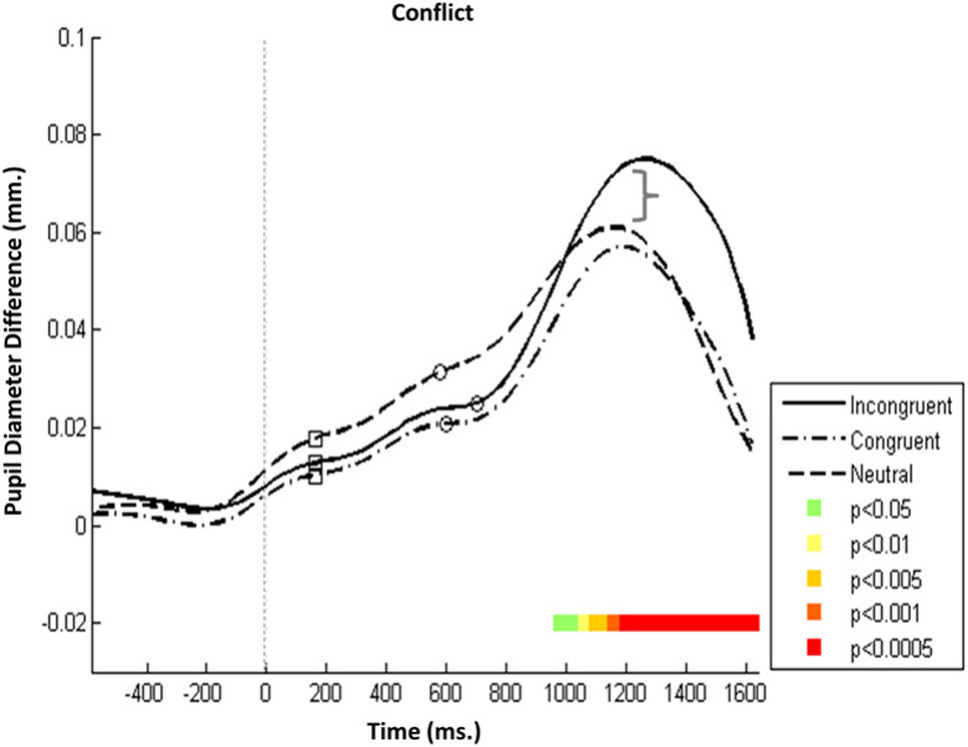
**Executive attention network—Mean PD dilation**. Blue line, incongruent flankers trials; black line, congruent flankers trials; cyan line, neutral flankers trials; white squares, mean entrance to target AOI; white circles, mean response time. Time zero represents target onset (dashed gray line) and −500 ms cue onset (dotted gray line).

In the executive network (Figure 4), the specificity of Pa and Pe to increased load was tested using three conditions: *a congruent condition*, in which all stimuli were compatible and congruent; *an incongruent condition*, in which the target cue was both incompatible and incongruent; and *a neutral condition*, in which the target cue was incompatible but not incongruent (Figure 1, upper left panel).

Analysis revealed that Pa was insensitive to the different conditions. On average, it was evoked at the same time and with the same amplitude in all three conditions (despite the seemingly slight elevation in the neutral condition). Conversely, Pe's amplitude was augmented in the incongruent condition. The increased dilation in the incongruent conditions became significant 1100 ms after target onset (Figure 4, *p* < 0.0005). Pe was not affected by perceptual incompatibility between stimuli (present both in the neutral and incongruent conditions), but it was sensitive to incongruency, which was only present in the incongruent condition. That this amplitude discrepancy in Pe is most noticeable in the executive network supports the notion that Pe is not related to stimuli processing but rather reflects the amount of effortful control invested in monitoring responses that involve cognitive load.

### Network-specific PD responses: peak amplitude and temporal characteristics

To characterize specific phasic pupillary changes as a function of ANT, differences in peak amplitudes of PD components (Pa and Pe) were analyzed using a repeated measures analysis first as a function of a specific network (alert, orient, or executive) and condition (network-baseline vs. network-specific cue). The results are summarized in Table 1.

**Table 1:** **Repeated measures analysis of PD components: Pa and Pe as a function of attention network and conditions**.

**Factor**	**Wilks′ Lambda F**	**Sig.**	**Partial η^2^**	**Within-subject contrasts**			
				**Significant contrast F**		**Sig.**	**Partial η^2^**
Component amp (Pa—Pe)	17.733	0.001	0.578	Linear	17.773	0.001	0.578
Condition (baseline—net Cue)	12.380	0.004	0.488	Linear	12.380	0.008	0.488
Network	7.380	0.008	0.550	Quadratic	15.737	0.002	0.548
Component × Condition	19.114	0.001	0.595	Linear	19.114	0.011	0.595
Component × Network	6.764	0.011	0.530	Linear	13.567	0.001	0.511
Network × Condition	3.554	0.061	0.372	Linear	6.594	0.023	0.337
				Quadratic	5.258	0.039	0.280
Network × Condition × Component	0.713	0.510	0.106				

Repeated measures analysis of variance (ANOVA) indicated three main effects (component amplitude, condition, and network) as well as two interaction effects (component × condition and component × network), such that Pe had larger amplitudes than Pa. These differences increased as a function of condition within networks (*p* < 0.001) and as a function of network in both a linear fashion (*p* < 0.001) and a quadratic fashion (*p* < 0.039). *Post-hoc* analyses revealed that differences in Pa amplitudes were greatest in the alerting network, and Pe differences were only observed in the executive control network.

**Figure 5:**
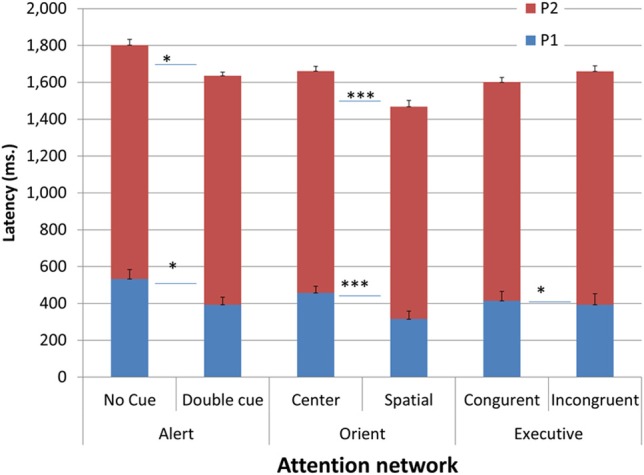
**Latencies of Pa and Pe onset as a function of condition and network**. ^*^p < 0.05, ^***^p < 0.001.

As for temporal characteristics, latencies of Pa and Pe onset as a function of condition and networks are presented in Figure 5. Repeated measures analysis showed that Pa, and consequently Pe, were shortest in the orienting spatial condition, which supports the acceleration hypothesis. Pa was also slightly shorter in the alerting double cue and executive incongruent trials relative to their respective baseline conditions; however, Pe latency was not shortened in the executive network, supporting an expected additional cross-network effect in the temporal dimension.

### Load-specific PD responses

Testing of limits of Pe as a function of accuracy and practice was conducted to examine the hypothesis that load specifically affects Pe but not Pa. These effects, which were expected to be strongest in error trials and in in-practiced trials, were supported.

Overall, error rates in the task were very low (1.8%), and most occurred in incongruent trials, with hardly any observed during congruent trials (98.42 ± 0.02%, *p* < 0.05 vs. 99.07 ± 0.02%, η^2^ = 0.328). Incidentally, the mean Pe amplitude in error trials as compared with correct incongruent trials was on average three times higher than in correct trials (0.3069 ± 0.14 vs. 0.0813 ± 0.05, *t* = 9.090, *p* < 0.001, respectively).

Secondly, to evaluate practice effects on Pa, Pe, accuracy, and RT, the 288 ANT trails were divided into three sections of 96 trails each, comprising the *least* practiced section, the *moderately* practiced section, and the *most* practiced section.

**Figure 6:**
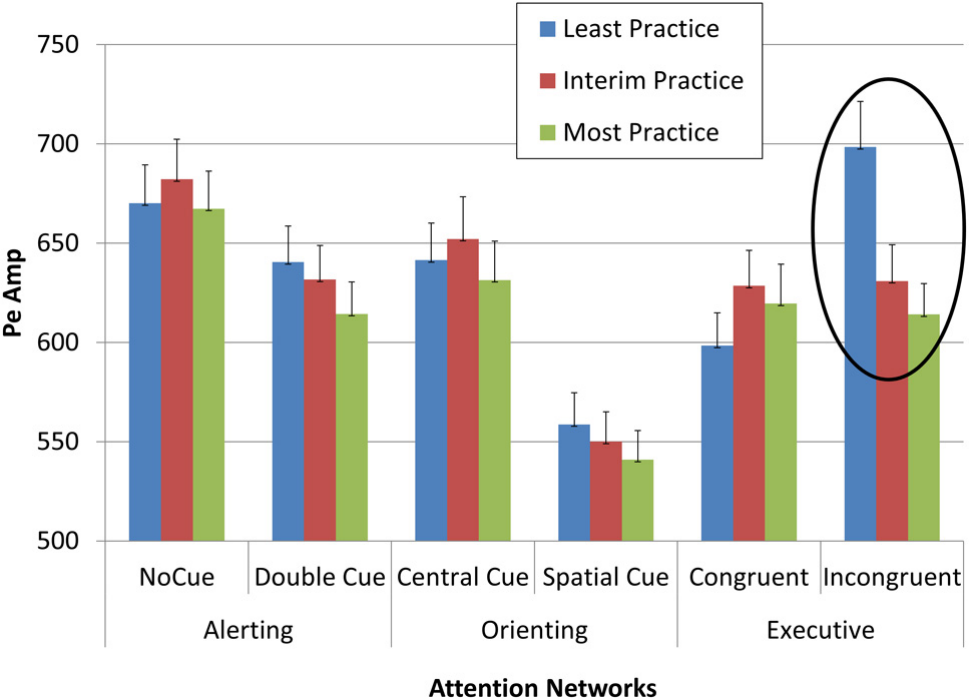
**Pe amplitude as a function of network, condition, and practice level**.

Multivariate ANOVAs with repeated measures for network, condition, and section effects on Pa, Pe, and RT showed that Pe was affected by practice (Table 2). The effect was particularly pronounced for Pe in the least practiced trials of the executive incongruent condition, where processing load is most pronounced (Figure 6).

**Table 2:** **Repeated measures analyses of pupillary and behavioral measures as a function of network, condition, and section**.

**Effect**	**Pa**			**AOI**			**RT**			**Pe**		
	**Wilks′ Lambda F**	**Sig.**	**Partial η^2^**	**Wilks′ Lambda F**	**Sig.**	**Partial η^2^**	**Wilks′ Lambda F**	**Sig.**	**Partial η^2^**	**Wilks′ Lambda F**	**Sig.**	**Partial η^2^**
Network	6.418	0.006	0.339	59.248	0.001	0.826	23.619	0.001	0.654	540.187	0.001	0.977
Condition	1.225	0.279	0.045	311.495	0.001	0.923	87.372	0.001	0.771	1118.382	0.001	0.977
Practice section	3.084	0.064	0.179	1.431	0.258	0.103	6.115	0.007	0.328	551.454	0.001	0.978
Network × condition	13.209	0.001	0.514	141.326	0.001	0.919	95.859	0.001	0.885	581.763	0.001	0.979
Network × section	0.278	0.889	0.046	0.646	0.635	0.101	8.650	0.001	0.601	251.351	0.001	0.978
Condition × section	1.098	0.351	0.080	0.750	0.483	0.057	6.664	0.005	0.348	541.722	0.001	0.977
Network × condition × section	0.976	0.440	0.145	1.924	0.141	0.251	13.750	0.000	0.705	255.315	0.001	0.978

The effect sizes of practice effects become evident by comparing the different sections. Unlike the factors of network and condition, to which all measures were sensitive, only some measures were affected by practice effects (Table 2). Specifically, Pa and on gaze entry to AOI were not affected by practice, its effect on RT was weak to moderate (0.33), and the practice effect on Pe was stronger than 0.90. These marked differences in effect size may point to a specific role for Pe in investing effort in monitoring, such that as the level of practice increases, the amplitude of Pe decreases, signifying that less effort is needed to maintain near perfect accuracy performance in high-load tasks that entail a risk of errors.

## Discussion

The current study contributes to existing attention regulation literature in three ways:

1. Highlighting specific PD activity in all attention networks: Using the attention network framework, it was evident that PD is evoked in each attention network in a construct-specific manner.
2. Proposed integrated hypothesis for PD in attention functions: The data are comparable with the gain-theory PD activation hypothesis, whereby alerting is related to Pa, an exploration LC mode. This initial component is accelerated by orienting to a specific cue in space and is followed by a later surge in PD (Pe) that corresponds with the recruitment of mental resources required to monitor performance and limit errors.

Overall, the data support a unique interplay between cognitive and autonomic noradrenergic reactions. They characterize *each* of the three attention networks by spatiotemporal differences in Pa and Pe.

Specifically, our analysis showed that Pa was evoked around 360 ms after a non-specific alerting cue but was not evoked in the absence of a cue. A similar finding was reported with skin conductance responses, which were recently proposed as a marker of LC-NE alerting activity (Murphy et al., 2011). Interestingly, its latency corresponds with an early event-related potential (ERP) detected during ANT performance (Neuhaus et al., 2010). Pa seems to reflect activation of autonomic changes necessary for supporting alerting and sustained engagement with stimuli-response contingencies necessary for learning.

Assessing the temporo-spatial characteristics of Pa enables a refinement of our understanding of autonomic function in the alerting network compared with the orienting network. Pa initiation latency and its acceleration rate were cue dependent, such that orienting cues to a particular location in space elicited accelerated Pa initiation.

The second PD component (Pe) was response locked; it was typically evoked 600 ms after the cue, and its latency to peak was around 900 ms after gaze was directed to the target. Its latency to peak corresponds with that recently reported using the Simon task (Van Steenbergen and Band, 2013). Pe's amplitude was prominent relative to the early Pa component and was particularly augmented during incongruent task processing (Figure 4). This is in line with the concept that executive monitoring task components are reflected in Pe and that the amplitude reflects the degree of effort necessary to manage load by inhibiting background noise and/or predominant response tendencies (Laeng et al., 2011), as well as response evaluation, adaptation (Van Steenbergen and Band, 2013), and cortical updating (Sokolov, 1963; Howells et al., 2012).

The temporal dimension in PD activation supports CL exploration-exploitation hypothesis of the pupillary response as it affects all three attention networks and facilitates understanding of the relationships between autonomic reactivity and voluntary regulation of motor activity. A typical progression is expected to be evidenced by early Pa onset, occurring about 300 ms after the cue (if perceived) and representing the recruitment of autonomic resources required for alerting and covert attention shifts in preparation of coding based on activity in the posterior attention system. This is followed by an overt gaze directed to the target, initiation of an action set, and a manual response to the target, which in turn activates the onset of a marked surge in PD (Pe) that is effort/reward dependent and seems to be decision locked. This is compatible with LC studies in monkeys indicating that the LC phasic response is driven by decision-making processes that serve to facilitate the behavioral response once a decision has been made (Clayton et al., 2004). Indeed, Pe's delayed activation and its prominent amplitude, particularly in conditions marked by incongruency, error trials, and least-practiced trials, is compatible with previous work with other tasks (Beatty, 1982) in a manner that seems to support the hypothesis that Pe is sensitive to the degree of invested effort and that it has a role in recruiting mental resources required for post-production executive monitoring and preparatory processes for on-going, high-load tasks (Lorist et al., 2000). Pe spatiotemporal characteristics may reflect inputs from the dorsolateral and ventromedial prefrontal systems of the anterior attention system and the ACC (Kennerley and Walton, 2011), which are needed for activating feedback loops to enable monitoring, inhibition, and reward regulation modulation that allow the inference of meaning, recognition, awareness, and learning.

Taken together, it seems that Pe reflects a surge in LC-NE through ACC top-down regulation by sufficiently increasing alertness for conflict-monitoring in a manner that would serve to drive a form of enhanced effortful control in future trials (Botvinick, 2007). Collectively, our results advance differential characteristics of specific attention functions; provide non-invasive quantifiable markers for alerting, orienting, and executive control monitoring; and attest to the versatility of pupillary activity in these vital faculties.

Future research may deepen the understanding of the role of the LC-NE network in the inter-relations among the attention networks and how arousal and orienting support/dampen executive attention (Mesulam, 1990; Van Steenbergen and Band, 2013). Such developments may further advance our knowledge regarding the roles of PD in primary learning-related constructs, such as processes of adaptation and generalization.

### Conflict of interest statement

The authors declare that the research was conducted in the absence of any commercial or financial relationships that could be construed as a potential conflict of interest.
